# Development and Internal Validation of a Predictive Model Including Pulse Oximetry for Hospitalization of Under-Five Children in Bangladesh

**DOI:** 10.1371/journal.pone.0143213

**Published:** 2015-11-18

**Authors:** Shahreen Raihana, Dustin Dunsmuir, Tanvir Huda, Guohai Zhou, Qazi Sadeq-ur Rahman, Ainara Garde, Md Moinuddin, Walter Karlen, Guy A. Dumont, Niranjan Kissoon, Shams El Arifeen, Charles Larson, J. Mark Ansermino

**Affiliations:** 1 Centre for Child and Adolescent Health, International Centre for Diarrhoeal Disease Research, Bangladesh, Dhaka, Bangladesh; 2 Centre for International Child Health, British Columbia Children’s Hospital, Vancouver, British Columbia, Canada; 3 The Department of Anesthesiology, Pharmacology & Therapeutics, The University of British Columbia, Vancouver, British Columbia, Canada; 4 The Department of Statistics, The University of British Columbia, Vancouver, British Columbia, Canada; 5 The Department of Electrical & Computer Engineering, The University of British Columbia, Vancouver, British Columbia, Canada; 6 The Department of Pediatrics, The University of British Columbia, Vancouver, British Columbia, Canada; Université Paris Descartes ; AP-HP, Groupe Hospitalier Cochin-Saint-Vincent-de-Paul, FRANCE

## Abstract

**Background:**

The reduction in the deaths of millions of children who die from infectious diseases requires early initiation of treatment and improved access to care available in health facilities. A major challenge is the lack of objective evidence to guide front line health workers in the community to recognize critical illness in children earlier in their course.

**Methods:**

We undertook a prospective observational study of children less than 5 years of age presenting at the outpatient or emergency department of a rural tertiary care hospital between October 2012 and April 2013. Study physicians collected clinical signs and symptoms from the facility records, and with a mobile application performed recordings of oxygen saturation, heart rate and respiratory rate. Facility physicians decided the need for hospital admission without knowledge of the oxygen saturation. Multiple logistic predictive models were tested.

**Findings:**

Twenty-five percent of the 3374 assessed children, with a median (interquartile range) age of 1.02 (0.42–2.24), were admitted to hospital. We were unable to contact 20% of subjects after their visit. A logistic regression model using continuous oxygen saturation, respiratory rate, temperature and age combined with dichotomous signs of chest indrawing, lethargy, irritability and symptoms of cough, diarrhea and fast or difficult breathing predicted admission to hospital with an area under the receiver operating characteristic curve of 0.89 (95% confidence interval -CI: 0.87 to 0.90). At a risk threshold of 25% for admission, the sensitivity was 77% (95% CI: 74% to 80%), specificity was 87% (95% CI: 86% to 88%), positive predictive value was 70% (95% CI: 67% to 73%) and negative predictive value was 91% (95% CI: 90% to 92%).

**Conclusion:**

A model using oxygen saturation, respiratory rate and temperature in combination with readily obtained clinical signs and symptoms predicted the need for hospitalization of critically ill children. External validation of this model in a community setting will be required before adoption into clinical practice.

## Introduction

In 2013, an estimated 6.3 million children died worldwide before their 5^th^ birthday [1]. Approximately 50% of these deaths are due to infectious causes that may be preventable if detected early and treated with simple cost effective therapies such as appropriate antibiotics and oral or intravenous fluids. The lack of clinical expertise for early and accurate diagnosis of serious infection in children less than 5 years of age, especially in community settings, is a significant roadblock to reducing mortality [2]. Integrated community case management (iCCM) is an attempt to address this issue. It is a strategy to train, support, and supply community health workers to diagnose and treat sick children of families with difficult access to case management at health facilities. However, even in the developed world, deaths from bacterial infection may be associated with a delay in seeking care, under evaluation of severity, and delayed antibiotic administration [3].

The iCCM can reduce mortality in diseases such as pneumonia by up to 70% [4]. However, case finding in the community is essential as it has been estimated that over 80% of deaths in children with pneumonia occur outside of facilities [5]. There is increasing evidence that large scale implementation of iCCM can reduce childhood mortality in diverse clinical contexts [6]. iCCM diagnosis is based on signs and symptoms that are designed to have a high degree of sensitivity in order to avoid missing any seriously sick child. However, the high sensitivity of the iCCM algorithms may result in high rates of referral of non-severe cases, with an increased burden on already under resourced and weak health care systems. An objective, point of care tool that would accurately identify children at higher or lower risk would help refine decisions about case management, such as community case management or the need for referral for higher level supportive care. At the community level, the challenge is identifying children who are critically ill as soon as possible to allow time for them to reach the nearest health facility.

Of particular interest in this study is the added predictive value of oxygen saturation (SpO_2_). Monitoring SpO_2_ with pulse oximetry in resource-poor settings, when coupled with a reliable oxygen supply has been shown to reduce mortality from pneumonia by as much as 35% [7,8,9]. In addition, a single estimate of SpO_2_ has been shown to be a significant predictor of radiological pneumonia in children [10]. However, the clinical utility of SpO_2_ as an isolated measurement is hampered by the lack of a clear threshold limit and the lack of availability of pulse oximetry for assessment of the illness severity in children in the community [11]. SpO_2_ in combination with other clinical signs and symptoms has been used to predict the need for admission to hospital in children with bronchiolitis [12], asthma [13] and pneumonia [14] in high resource settings and as a prognostic predictor of mortality in low resource settings [9,15,16].

The purpose of this study was to develop and internally validate a simple model that would be predictive of the need for admission to a health facility using clinical signs and symptoms in combination with oxygen saturation and heart rate measured by pulse oximetry. We chose to study children presenting at this facility, rather than in the community, to ensure that we would have reliable assessments performed by the experienced facility physicians. The practicality of collecting a large enough sample size and the need for good outcome data dictated this decision. Moreover, this tertiary hospital was the only health resource for the catchment population.

## Methods

### Ethics Statement

Institutional review board approval was obtained from the Research Review Committee (RRC) and Ethical Review Committee (ERC) of the Institutional Review Board of International Centre for Diarrhoeal Disease Research, Bangladesh and the University of British Columbia/Children’s and Women’s Health Centre Research Ethics Board. We explained the study and obtained written informed consent from the caretaker accompanying the child.

### Study Design and Population

We conducted a prospective observational study at the Kumudini Women’s Medical College Hospital’s (KWMCH). KWMCH is a not-for-profit private tertiary level hospital located in the Mirzapur sub-district of Tangail district in Bangladesh, with more than 12,000 annual outpatient visits for children less than 5 years of age. KWMCH provides primary level care to a catchment population of approximately 500,000 people.

All children less than 5 years of age presenting at the outpatient department or emergency department from October 2012 to April 2013 (winter is from November to February) were eligible for inclusion in this study. We assessed the first presenting case of a day followed by the next eligible case in the queue after completion of the preceding assessment. Children were seen based on time of arrival and no triage process occurred before cases were selected. We excluded children presenting with chronic diseases or those who had documented low SpO_2_ levels due to other conditions, such as cardiac disease.

The attending hospital physician undertook a complete history and physical examination and made a treatment recommendation. Our study physicians then extracted data from the hospital medical record and documented all demographic data, clinical signs, and symptoms such as weight, respiratory rate, and temperature in a structured case report form. In addition, the study physicians performed a one minute recording of the photoplethysmographic (PPG) waveform and extracted SpO_2_ and heart rate (HR) using an Xpod^®^ external original equipment manufacturer (OEM) pulse oximeter (Nonin Medical Inc., Plymouth, USA; model 3012LP) connected to a mobile device the 4^th^ generation iPod touch (Apple Inc., Cuppertino, USA) and a custom data collection app (‘PhoneOxR2’ version 2.2.3 [17]). The interface of the PhoneOxR2 application displayed the PPG waveform with a color coded (8 shades of green to red) background based on the calculated signal quality index (SQI). The SQI was calculated using the amplitude of the PPG and variability of the SpO_2_ and HR and pulse oximeter module generated alerts. The research physicians were trained to optimize the SQI for data quality purposes. The recorded SpO_2_ was kept hidden from the facility and study physicians, as the SpO_2_ measurement was not routinely available in this clinical setting. The SpO_2_ reading was not used for making any clinical decisions and was only available during analysis of the data. The application also included the measurement of respiratory rate by tapping the screen of the mobile device with each breath [18]. This application was built using Lambda Native [19], a cross-platform open-source development environment written in Scheme. In PhoneOxR2, physiological trends from PPG were recorded to a comma-separated-value file at 1 Hz and the PPG waveform was recorded at 75 Hz. No decisions on hospitalization were made based on the research data collection.

The clinical course of all hospitalized and non-hospitalized children was followed and documented. Children who were sent home from the outpatient facility or emergency department were followed-up with a telephone call 96 to 120 hours after their initial visit at the facility.

### Data Quality Management

All case record forms were entered using double data entry and checked for completeness and accuracy. Validation rules such as range check, uniqueness check, and skip rules were used with the data entry interface to verify the consistency of data. The PhoneOxR2 software provided automatic encryption and synchronization through a wireless connection and Application Programming Interface (API) to a web-based data collection system (REDCap) [20].

### Statistical Analysis

Statistical analyses were performed using STATA/SE 12.1 (StataCorp LP, College Station, USA) and R (3.1.0) [21]. The primary outcome for the analysis was the need for hospitalization for ongoing care. Admission was considered necessary if children were admitted and stayed for more than 24 hours in the hospital, were sent home initially but admitted at a subsequent visit within 1 week, were advised for admission but the advice was not followed, or were transferred or referred to another facility providing a higher level of care for admission. We considered all children who were sent home or admitted and discharged within 24 hours as not having illness of sufficient severity to require hospitalization for ongoing care.

### Candidate Variable Selection

The candidate predictor variables were selected based on a literature review, *a priori* consensus of clinical importance, prevalence in the study population and ease of measurement in resource-poor settings. The relationship between predictor variables and the outcome was assessed using univariate logistic regression using odds ratio [OR] (with confidence intervals). All continuous variables were assessed for co-linearity. Nonlinear predictors were transformed using logarithmic, exponential or square transformations, based on the pattern of nonlinearity. A weight-for-age z-score was calculated. A physiological transformation based on the shape of the relationship between SpO_2_ and virtual shunt [22] was used to linearize the SpO_2_ measurements [70*log10(104-SpO_2_)-57]. This virtual shunt was used as an index of disease severity. To evaluate the added value of pulse oximetry, we calculated the net reclassification improvement (NRI) and integrated discrimination improvement (IDI) of the model with SpO_2_ [23].

To reduce co-linearity, high multi-co-linearity, as measured by correlation coefficient (-0.9 < corr < +0.9), and variable inflation factor (≥ 10.0) between variables was determined and only the more clinically relevant variable of a pair of highly correlated variables was retained. The majority of the categorical variables were found to be highly correlated. When a high degree of correlation existed between two categorical variables they were re-coded as a combined indicator variable when possible, but were otherwise retained in the model, if considered important in the outcome prediction.

### Missing Values

Predictor variables with a low number of missing values (< 10 cases missing) had these cases excluded. The SQI of the recorded PPG waveform was low in a number of children and was considered clinically related to both the age and disease severity of the child. A new variable based on the SQI and an estimate of virtual shunt from the SpO_2_ was included as a candidate predictor [21]. Missing data in weight for age z-scores were calculated based on imputed values from age and sex variables. The missing values for weight, respiratory rate and temperature were imputed using a multiple imputation approach [24] to avoid potential bias introduced by ignoring missing data uncertainty. Each missing value was imputed using five plausible values, leading to five complete datasets. As the rate of missing information was low, five imputations were considered adequate [25]. Standard logistic regression analysis was conducted for each of the five complete datasets. The results were later pooled to yield estimates, confidence intervals and p-values that incorporated missing data uncertainty. Association between the primary outcome (i.e., cases requiring admission), and each of the clinical signs and symptoms was examined in a series of univariate analyses.

### Model Building

A series of multivariate logistic regression models were incorporated to identify the individual variables that predicted the primary outcome. A stepwise selection approach was applied, starting with an empty model and adding or removing one predictor at each step. Models were compared using the BIC (Bayes Information Criterion), AIC (Akaike Information Criterion) and LASSO (Least Absolute Shrinkage and Selection Operator) methods. Further inclusion into the list of variables was made based on clinical knowledge. The Hosmer-Lemeshow goodness-of-fit test was conducted to evaluate the calibration of the final model.

The sample size required for model development was determined on the basis of the minimum standard of 10 events per effective variable considered in the model according to the formula N = (n*10)/I where N is the sample size, n is the number of candidate predictor variables, and I is the estimated event rate in the population [26]. An estimated event rate of 20% was used for a model with 39 effective candidate predictor variables. A minimum of 1950 children were estimated to be required. It was estimated that a 6 month data period would provide a significantly large sample size.

### Internal Model Validation

The bootstrap procedure was employed for internal validation [27]. The predictor selection was applied to each bootstrap sample to obtain a final model, and the optimism was estimated by comparing the final model performance to the original data for each bootstrap sample. The bootstrap corrected area under the curve was computed by subtracting the optimism from the original area under the curve.

### Model Performance

To assess the calibration capability of the model to identify children who required admission to a facility, we used crossover testing on the same sample set to test if the prediction rule successfully predicted individual case requirements. We calculated the classification performance (sensitivity, specificity, positive and negative predictive values, and positive and negative likelihood ratios) for each possible risk threshold. A risk stratification table was used to assess the distribution of the outcome in each risk category stratified by the model prediction [28]. We selected a range of possible thresholds to identify the trade-off between identifying sick children and unnecessary admission of cases that could be used in different clinical contexts. A weighted classification score, (the number of correct identifications of true positives) + (the number of correct identifications of true negatives), was calculated using various ratios of false negative cases to false positive cases (1:3; 1:5 and 1:10) and plotted to identify important inflection points. The final threshold was chosen to maximize the weighted classification score.

## Results

**Fig 1:**
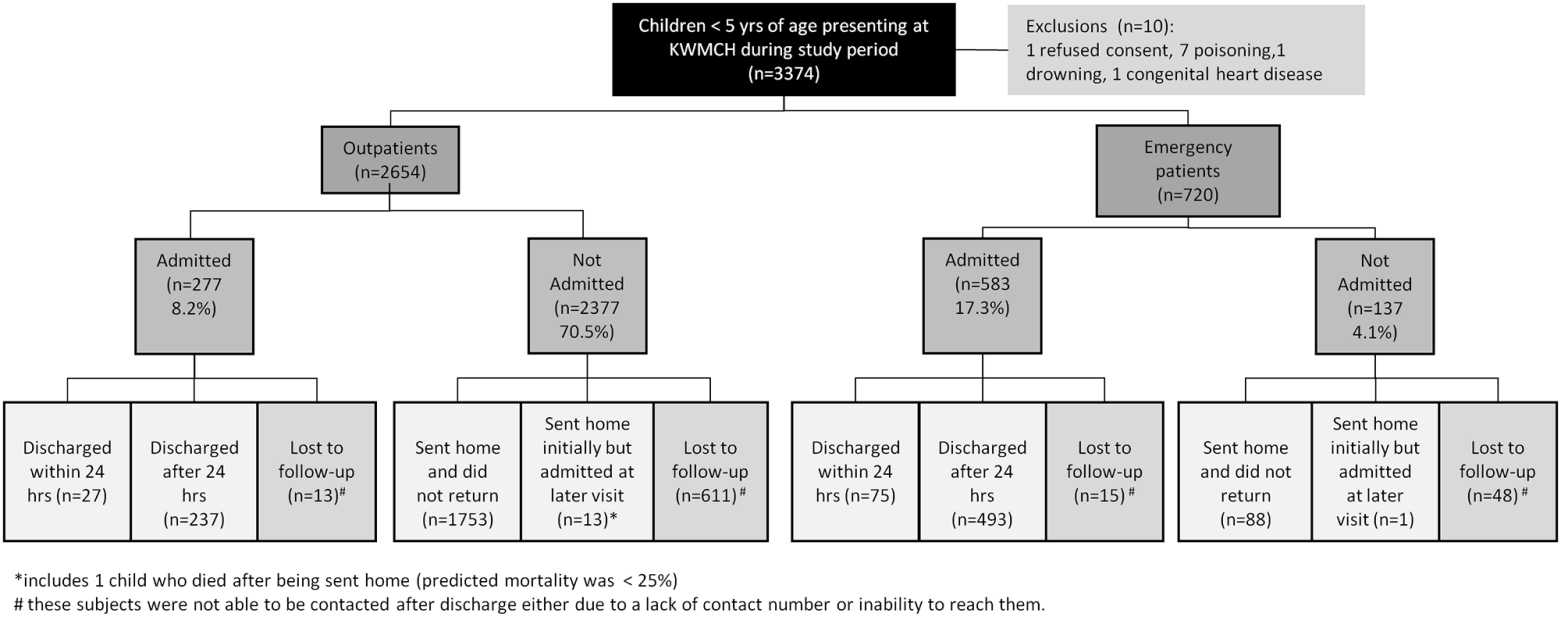
Flowchart of study population and distribution of outcomes.

We enrolled 3374 children less than 5 years of age in the study. The median (interquartile range) age was 1.02 (0.42–2.24) years and 62% were male. Twenty-five percent were admitted and 102 of these children (11.9%) were discharged within 24 hours. Thirteen (< 1%) children who were sent home following the initial visit were subsequently admitted to KWMCH or elsewhere. One child died at home and 687 (20%) were lost to follow-up (Fig 1). The children lost to follow- up were on average older, had lower weight for age z-scores, a higher prevalence of fever and a lower prevalence of diarrhea. No other systematic differences were found between the children lost to follow-up and those that had been followed-up (see S1 File for more details).

The most common diagnoses in children requiring admission were acute lower respiratory tract infections (40.6%), and acute upper respiratory or eye or ear infection (20.1%). In children discharged home, the most common diagnosis was acute upper respiratory or eye or ear infection (50.4%), diarrhea (16.1%), fever of unknown cause (14%) and other gastrointestinal complains (8.7%). The prevalence of acute lower respiratory infection in children not requiring admission was very low (1.2%) (Table 1).

**Table 1:** Primary diagnosis reported by facility physician for children included in the study*.

Diagnosis Type	Study criteria for admission not fulfilled (n (%))	Study criteria for admission fulfilled (n (%))
Acute upper respiratory/eye/ear infection	1224 (50.4%)	190 (20.1%)
Diarrhea	391 (16.1%)	46 (4.9%)
Acute lower respiratory infection	29 (1.2%)	384 (40.6%)
Fever of unknown cause/Typhoid	340 (14%)	36 (3.8%)
Other gastrointestinal symptoms	211(8.7%)	6 (0.6%)
Reactive airway disease	66 (2.7%)	53 (5.6%)
Skin/umbilical infection	27 (1.1%)	49 (5.2%)
Convulsion/Epilepsy	7 (0.3%)	36 (3.8%)
Vomiting	34 (1.4%)	1 (0.1%)
Others	68 (2.8%)	118 (12.5%)
Missing	32 (1.3%)	26 (2.7%)
Total number of children	2429	945

*Admission was considered necessary if children were admitted and stayed for more than 24 hours in the hospital, were sent home initially but admitted at a subsequent visit within 1 week, were advised for admission but the advice was not followed, or were transferred or referred to another facility providing a higher level of care for admission.

### Univariate Analysis

Children who required admission were younger, had lower median weight for age z-scores and SpO_2_ values, and had higher likelihood of breathing difficulty, fever, cough, chest in-drawing and lethargy compared to children who did not require admission. Children who were sent home were more likely to have symptoms such as diarrhea and vomiting although these were more likely to be present for more than 24 hours in those requiring admission. The univariate analysis with lethargy, low SpO_2_ or fast breathing revealed an area under the receiver operating characteristic curve (AUC) of over 0.70 (Table 2).

**Table 2:** Predictor variable distribution and odds ratio for study population^#^.

Input Predictors	Study criteria for admission not fulfilled	Study criteria for admission fulfilled	Univariate AnalysisOdds Ratio (95% CI)
Age (days) ^a^(n = 3374)	427 (204 to 913)	230 (76 to 537)	0.9989* (0.9987 to 0.9991)
Male (%)(n = 3374)	60.4	65.9	1.271* (1.086 to 1.487)
Weight-for-age z-score (n = 2943) ^a^	-0.7 (-1.7 to -0.2)	-0.9 (-1.9 to -0.1)	0.910* (0.864 to 0.957)
Heart rate (beats/min) (n = 3368) ^a^	129 (115 to 143)	141 (127 to 156)	1.028* (1.024 to 1.031)
Respiratory rate (breaths/min) (n = 3309) ^a^	33.0 (28.7 to 39.0)	40.9 (33.2 to53.6)	1.078* (1.069 to 1.086)
Temperature (°C) (n = 3360) ^a^	37.0 (36.8 to 37.1)	37.0(37.0 to 37.6)	2.057* (1.834 to 2.308)
Fever (%)	28.8	42.2	1.809* (1.547 to 2.114)
Fever >24 hrs (%)	21.0	30.8	1.678 * (1.417 to 1.988)
Cough (%)	58.2	70.4	1.708* (1.453 to 2.006)
Cough >24 hrs (%)	53.5	64.0	1.548* (1.326 to 1.807)
Vomiting (%)	9.8	9.0	0.914 (0.705 to 1.186)
Vomiting >24 hrs (%)	6.2	7.4	1.225 (0.906 to 1.631)
Difficult or fast breathing (mother) (%)	6.7	52.0	15.035* (12.262 to 18.434)
Abdominal pain (%)	4.1	2.1	0.509* (0.313 to 0.828)
Diarrhea (%)	17.2	6.6	0.339* (0.257 to 0.448)
Diarrhea >24 hrs (%)	12.1	4.8	0.362* (0.262 to 0.500)
Chest in-drawing (%)	0.7	26.9	49.236* (30.300 to 80.006)
Difficult breathing (physician) (%)	1.6	21.4	16.237* (11.453 to 23.020)
Lethargy (%)	6.1	56.1	19.683* (15.953 to 24.285)
Irritability (%)	3.0	9.7	3.481* (2.535 to 4.780)
Oxygen saturation (%)^b^ (n = 3368)^a^	98 (97 to 99)	96 (92 to 98)	1.078* (1.070 to 1.086)
SQI (n = 3345)^a^	93 (88 to 95)	93 (88 to 95)	1.003* (0.998 to 1.008)

^**a**^ Distribution of continuous variables by outcome type is presented as median (inter-quartile range)

^b^ Oxygen saturation (SpO_2_) values are transformed using formula [70*log10(104-SpO_2_)-57] to derive OR

*p-value <0.05

SQI: Signal Quality Index

^#^Admission was considered necessary if children were admitted and stayed for more than 24 hours in the hospital, were sent home initially but admitted at a subsequent visit within 1 week, were advised for admission but the advice was not followed, or were transferred or referred to another facility providing a higher level of care for admission.

### Multivariate Analysis and the Final Model

BIC consistently identified the most parsimonious model. AIC and the LASSO methods did suggest a few additional predictors, however, their inclusion only marginally improved prediction. Therefore, the set of predictors identified by BIC were used. The predictor selection was repeated for each of the five complete datasets following imputation for missing variables (12.77% for weight, 1.04% for respiratory rate and 0.42% for temperature) and the same set of predictors were consistently identified in four of the five complete datasets.

**Fig 2:**
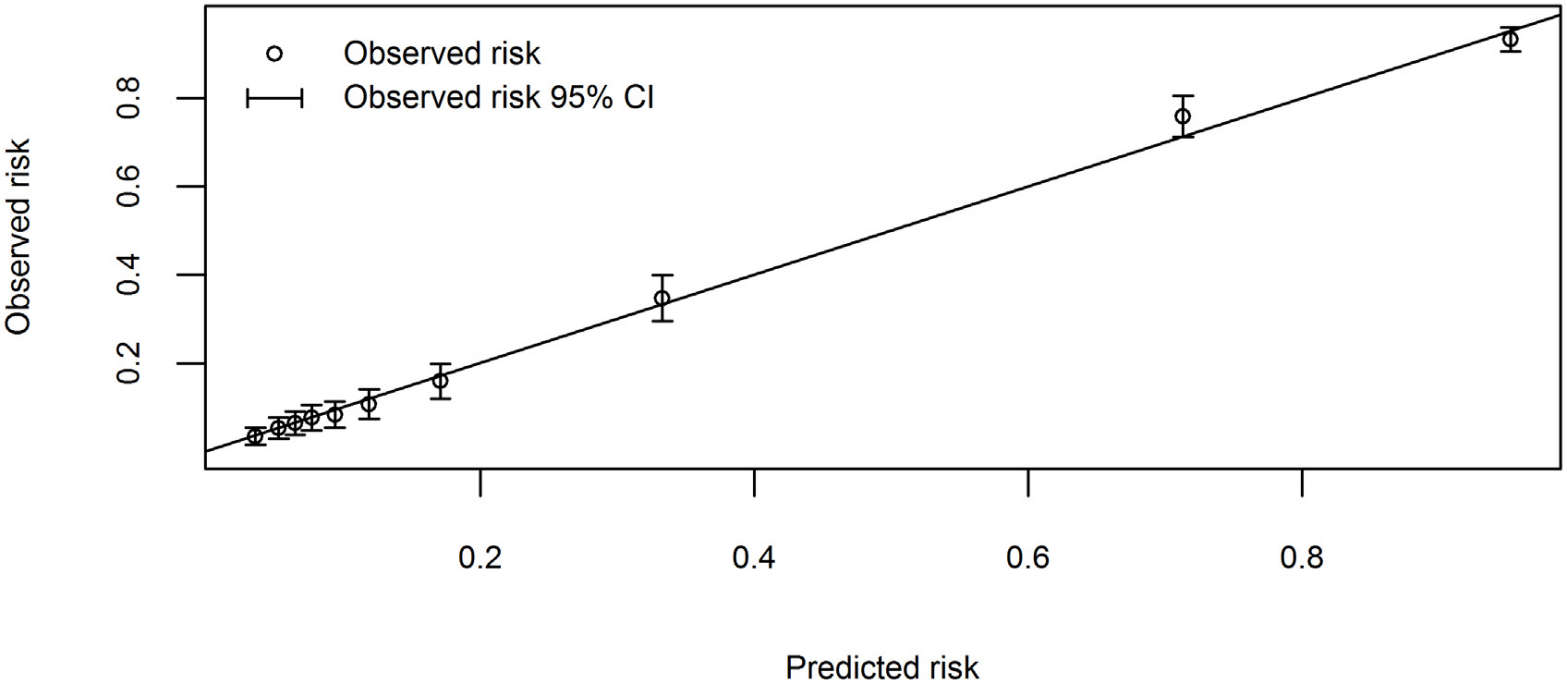
Receiver operating characteristic curve of the final model in the study cohort. AUC ROC = area under the curve of the receiver operating characteristic. Sens = sensitivity. Spec = specificity. PPV = positive predictive value. NPV = negative predictive value.

**Fig 3:**
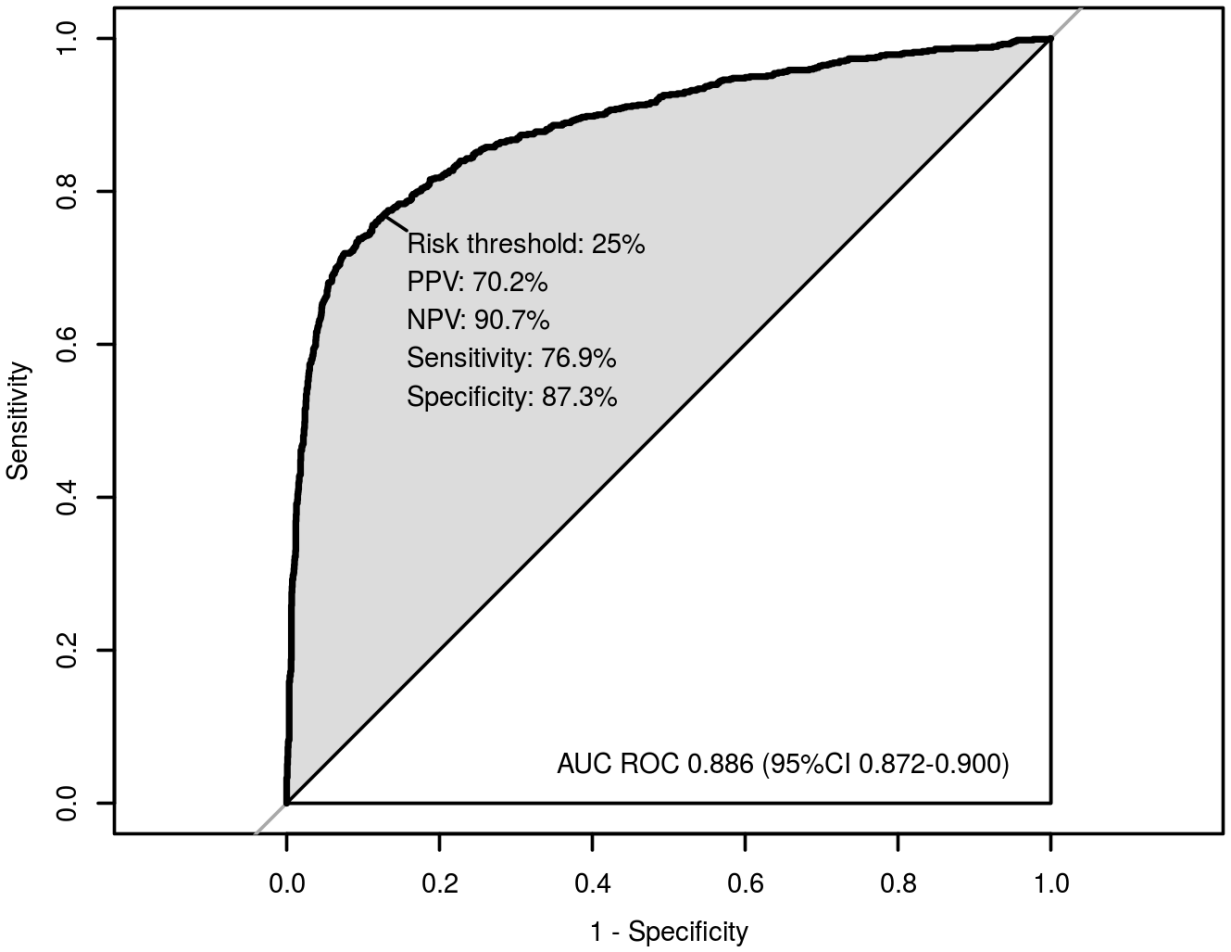
Calibration plot of the final 10-predictor model applied to the 3263 cases excluding subjects who were unconscious or who had experienced convulsions (Hosmer-Lemeshow goodness-of-fit p-value = 0.53). The 45 degree straight line corresponds to the line of perfect calibration on which model predicted risks coincide with the observed risks.

The final model was reduced to 10 predictor variables (Table 3). This final model had an AUC of 0.89 (95% CI: 0.87 to 0.90) (Fig 2). The Hosmer-Lemeshow goodness-of-fit test indicated evidence of lack of fit (p = 0.01), however, a closer look at the standardized residuals within each decile reveals that all discrepancies come from the 20% sickest cases, with the highest 20% of predicted admission probabilities. The exclusion of subjects with convulsions or who were unconscious significantly improved the fit (Fig 3).

**Table 3:** Adjusted odds ratios of predictor variables in the final prediction model.

Predictors	Coefficient(intercept = -18.05)	Odds Ratio (OR)	Confidence Interval (95%)	
			Lower	Upper
Age of child (in 100 days)	-0.05	0.95	0.93	0.98
Chest in-drawing	2.18	8.85	5.06	15.45
Cough	-0.86	0.42	0.33	0.54
Difficult OR fast breathing(reported by mother)	1.31	3.70	2.77	4.95
Diarrhea	-0.98	0.38	0.26	0.54
Irritability	1.09	2.99	1.97	4.53
Lethargy	2.19	8.90	6.91	11.46
Respiratory rate (breaths/min)	0.02	1.02	1.01	1.03
Oxygen saturation (%)[transformed]	0.02	1.02	1.01	1.03
Temperature (°C)	0.43	1.54	1.32	1.78

p-values associated with the adjusted odds ratios are all less than 0.0002.

The addition of SpO_2_ to the final predictors exhibited 7% net improvement in the classification of the non-admitted and a 4% net improvement in the classification of the admitted subjects, at a risk threshold of 25%. The IDI was 0.005 (95% CI: 0.002 to 0.007, p-value <0.001).

### Risk Prediction

**Fig 4:**
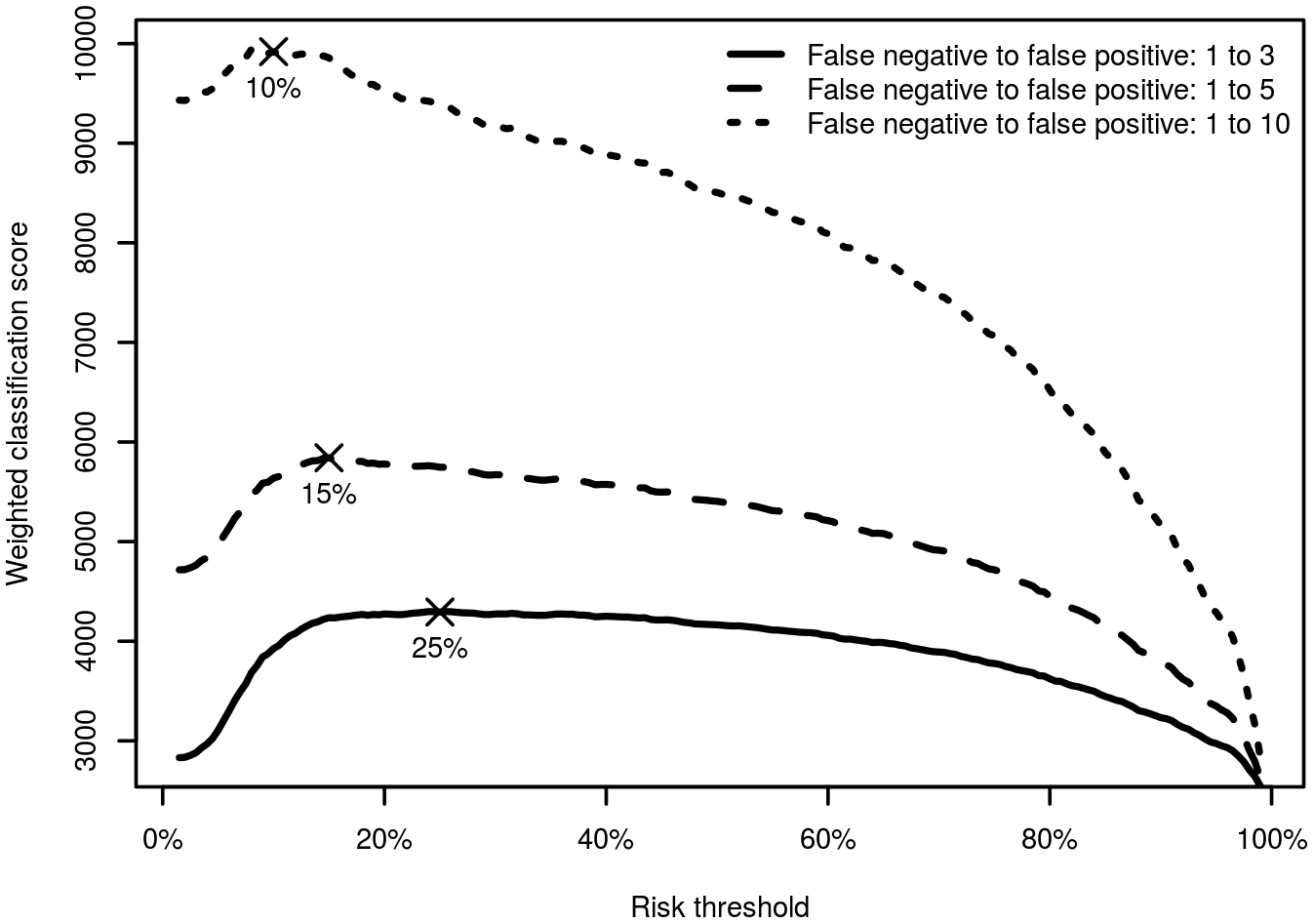
Weighted classification score for the full range of thresholds using different trade-offs between false negative and false positive cases.

At a risk threshold of 25% the model can correctly classify 77% of children requiring admission (sensitivity) and 87% of children not requiring admission (specificity), with positive predictive value (PPV) of 70% and negative predictive value (NPV) 91% (Fig 2). Lowering the risk threshold to 15% increases the sensitivity to 85% and decreased the specificity to 75%. The PPV decreases to 57% and NPV increases to 93% (Table 4 and Fig 4).

**Table 4:** Classification performance measures of final prediction model at different risk thresholds.

Levels of Predicted Probability of Admission	Admission Required (n/%)	Likelihood Ratio (95% CI)	Total/Percent Cases in Range
0–5%	13 (4%)	9.45 (5.46–16.38)	329 (9.8%)
5.1–10%	74 (6.6%)	5.48 (4.38–6.85)	1117 (33.2%)
10.1–15%	53 (10.2%)	3.43 (2.61–4.52)	521 (15.5%)
15.1–20%	50 (21%)	1.46 (1.08–1.98)	238 (7.1%)
20.1–25%	26 (21.1%)	1.45 (0.95–2.22)	123 (3.7%)
>25%	727 (69.9%)	0.17 (0.15–0.19)	1040 (30.9%)
Total	943 (28%)		3368

### Internal Validation

Each of the 10 final selected predictors was also selected in at least 80% of the bootstrap samples indicating a stable predictive capability. The bootstrap corrected AUC was 0.87 (95% CI: 0.86 to 0.89). The model stability was further ascertained to be consistent for each of the 5 imputed datasets. The details of internal validation can be found in S2 File.

## Discussion

### Summary of Findings

We have developed and internally validated a clinical prediction model to predict the need for hospitalization in children under 5 years of age presenting at an outpatient department. Breathing difficulty, chest in-drawing, irritability, lethargy, an increased temperature, oxygen saturation and respiratory rate were the strongest predictors of the need for hospitalization in line with previous studies and current international guidelines. The presence of cough was a positive predictor in the univariate prediction but cough and diarrhea were protective in predicting admission. A model with 10 predictors has provided an AUC of 0.89 (95% CI: 0.87 to 0.90) and at a risk threshold of 25% has PPV value 70% (95% CI: 67% to 73%) and NPV of 91% (95% CI: 90% to 92%).

### Importance of Study

This prediction model has concentrated on variables that could be readily measured in the community. Predictors that have been used by more experienced clinicians such as ‘gut feeling’ and chest auscultation were not considered [29]. In addition, the use of a pulse oximeter provides an objective measure of SpO_2_ and HR but would be dependent on the availability of the device in the community. The barrier of availability can be overcome by using a mobile app and low cost sensor attached to the same mobile device [30].

There is increasing evidence to suggest that mHealth (the use of mobile devices with software applications to provide health services and manage patient information) can be used to deliver increased and enhanced health care services to individuals and communities, while helping to strengthen health systems and retain community health workers [31]. The computing power and display capability of even the entry level smartphones in low resource settings provide the opportunity to repurpose these as medical diagnostic devices for community measurement of vital signs [32]. Mobile devices also provide a platform to implement clinical guidelines such as iCCM and prediction models in low resource settings, with low training and follow-up support overhead and at an affordable cost.

The inclusion of vital signs including SpO_2_ as continuous variables is a significant advance in the development of this prediction model. It has previously been demonstrated that using categorical variables in prediction models for HR and respiratory rate results in significant information loss and lower predictive ability [33]. The inclusion of multiple continuous vital sign measurements in the model allows us to capture the known clinical interactions between variables such as respiratory rate and age or temperature [34]. While a low SpO_2_ has been clearly demonstrated to predict both the need for hospitalization and mortality [35], many of these studies were performed at high altitude. The lower partial pressure of inspired oxygen at altitude moves the normal range of the SpO_2_ curve to the steeper and more linear slope of the S shaped dissociation curve [36, 37]. We have chosen to perform a transformation based on the physiological concept of virtual shunt [23]. This transformation will linearize the flat end of the oxygen curve and allows us to use a continuous variable for SpO_2_. The transformation overcomes the limitations of using a single threshold value for SpO_2_ such as that adopted by the World Health Organization (WHO) [38]. The barometric pressure available with many mobile devices will also allow us to correct this SpO_2_ variable for the change in inspired oxygen partial pressures when the prediction is used at different altitudes [8].

### Interpreting Risk

A prediction model is only clinically useful if it is able to support clinical decision making. At a threshold of 25% the model showed a 77% sensitivity, 87% specificity, 70% positive predictive value and 91% negative predictive value for need of hospital admission. The sensitivity can be further increased (88%) at a lower risk threshold but at a cost of a decrease in specificity (69%). In a similar clinical setting, the use of Integrated Management of Childhood Illness guidelines resulted in a sensitivity of 86% and specificity of 64% for predicting hospital admission [39]. There is always a trade-off between acceptable risk and undue burden to the family, patient and health system. A marginal score could be used to direct further reassessment of the child to prevent depriving them of potentially lifesaving interventions. The appropriate risk threshold and actions taken would depend on the specific local context such as transportation, local treatment options and family choice. For developing countries with limited resources and where the health system is already over burdened due to several supply side constraints [35], it is probably more appropriate to use a guideline with a higher specificity [40]. The lack of optimal sensitivity would mean that some children requiring admission would be sent home. Parents would need to be encouraged to return for further assessment even if they fell below the risk threshold and they were still concerned about the clinical condition of their child.

### Model Calibration

The lack of calibration of the model was not unexpected. During the predictor variable selection process we did identify certain danger signs, such as unconsciousness or convulsions, which would indicate the need for admission to a facility without the need to calculate a prediction score. The Hosmer-Lemeshow test is known to be sensitive to sample size when there is small deviation from the perfect fit. We believe that as the fit is poor only in the highest 20% of predicted admission probabilities, the model with 10 predictors provides an acceptable calibration for the 3374 eligible cases. If we remove the 105 (3%) cases with unconsciousness and convulsions, the derived final model still contains the same 10 predictors with similar coefficients, but with significantly improved calibration (p = 0.53).

### Limitations

There are a number of potential limitations of this study. The data was collected at a tertiary level facility rather than in the community. We would anticipate that the case mix would not be significantly different than that encountered in the community. This assumption will need to be validated in future studies. There was a risk that the facility physicians would be overly conservative in their decision making knowing that their clinical outcomes were being followed. To reduce this risk of bias in the model we excluded cases that were discharged within 24 hours, we did not provide the pulse oximetry data to the facility physicians and we used dedicated study physicians to collect the data. To avoid missing any cases that were inadvisably sent home we attempted to contact all families sent home, we specifically looked for children who were admitted at a subsequent visit and we documented the families who chose not to follow the advice for admission to the facility. However, these cases only constituted a small fraction of the total number of cases studied. There was significant variability in the diagnosis listed by the facility physician in the children that were not admitted to the facility, while the diagnoses of the children admitted to the facility were much easier to verify based on further investigations and the clinical course of the disease. There may have been children who improved very rapidly and warranted admission and early discharge based on a response to treatment. The decision to classify children based on these criteria was made a priori. There were a very low number (107) of children discharged within 24 hours.

## Conclusion

This simple model was able to predict which children were admitted to a health facility. However, the model requires prospective external validation before introduction into clinical practice. The use of SpO_2_ as a continuous variable with appropriate transformation based on well described physiological principles reduces the information loss produced by using a single threshold for classification of risk.

## Supporting Information

S1 File Characteristics of children lost to follow-up. (DOC)

S2 File Details of internal validation. (DOCX)

S3 File Original data. (CSV)

## References

[1] Liu L, Oza S, Hogan D, Perin J, Rudan I, Lawn J, et al Global, regional, and national causes of child mortality in 2000–13, with projections to inform post-2015 priorities: an updated systematic analysis. Lancet. 2015; 385 (9966): 430–40. 10.1016/S0140-6736(14)61698-6 25280870

[2] UNICEF. Committing to Child Survival: A Promise Renewed. 2012. UNICEF Progress Report 2012. Available: http://www.un.org/ru/publications/pdfs/renewed_%20progress%20report%202012.pdf

[3] Launay E, Gras-Le Guen C, Martinot A, Assathiany R, Martin E, Blanchais T, et al Why children with severe bacterial infection die: A population–based study of determinants and consequences of suboptimal care with a special emphasis on methodological issues. PLoS One. 2014; 9 (9): e107286 10.1371/journal.pone.0107286 25247401 PMC4172434

[4] Theodoratou E, Al-Jilaihawi S, Woodward F, Ferguson J, Jhass A, Ballliet M, et al The effect of case management on childhood pneumonia mortality in developing countries. Int J Epidemiol. 2010; 39(Suppl. 1):i155–71. 10.1093/ije/dyq032 20348118 PMC2845871

[5] Nair H, Simões EA, Rudan I, Gessner BD, Azziz-Baumgartner E, Zhang JSF, et al Global and regional burden of hospital admissions for severe acute lower respiratory infections in young children in 2010: a systematic analysis. Lancet. 2013; 6736(12): 4–6. 10.1016/S0140-6736(12)61901-1 PMC3986472 23369797

[6] Marsh DR, Hamer DH, Pagnoni F, Peterson S. Introduction to a special supplement: Evidence for the implementation, effects, and impact of the integrated community case management strategy to treat childhood infection. Am J Trop Med Hyg. 2012; 87(Suppl 5): 2–5. 10.4269/ajtmh.2012.12-0504 23136271 PMC3748517

[7] Duke T, Wandi F, Jonathan M, Matai S, Kaupa M, Saavu M, et al Improved oxygen systems for childhood pneumonia: a multihospital effectiveness study in Papua New Guinea. Lancet. 2008; 372(9646): 1328–1333. 10.1016/S0140-6736(08)61164-2 18708248

[8] Duke T, Subhi R, Peel D, Frey B. Pulse oximetry: technology to reduce child mortality in developing countries. Ann Trop Paediatr. 2009; 29(3): 165–75. 10.1179/027249309X12467994190011 19689857

[9] Subhi R, Adamson M, Campbell H, Weber M, Smith K, Duke T, et al The prevalence of hypoxaemia among ill children in developing countries: a systematic review. Lancet Infect Dis. 2009; 9(4): 219–27. 10.1016/S1473-3099(09)70071-4 19324294

[10] Modi P, Munyanezy RB, Goldberg E, Choy G, Shailam R, Sagar P, et al Oxygen saturation can predict pediatric pneumonia in a resource-limited setting. J Emerg Med. 2013; 45(5): 752–60. 10.1016/j.jemermed.2013.04.041 23937809

[11] Ginsburg AS, Van Cleve WC, Thompson MI, English M. Oxygen and pulse oximetry in childhood pneumonia: a survey of healthcare providers in resource-limited settings. J Trop Pediatr. 2012; 58(5):389–393. 10.1093/tropej/fmr103 22170511

[12] Walsh P, Rothenberg SJ, O’Doherty S, Hoey H, Healy R. A validated clinical model to predict the need for admission and length of stay in children with acute bronchiolitis. Eur J Emerg Med. 2004; 11: 265–272. 10.1097/00063110-200410000-00005 15359199

[13] Keahey L, Bulloch B, Becker AB, Pollack CV, Clark S, Camargo C, et al Initial oxygen saturation as a predictor of admission in children presenting to the emergency department with acute asthma. Ann Emerg Med. 2002; 40(September): 300–307. 10.1067/mem.2002.126813 12192354

[14] Van den Bruel A, Haj-Hassan T, Thompson M, Buntinx F, Mant D, et al Diagnostic value of clinical features at presentation to identify serious infection in children in developed countries: a systematic review. Lancet. 2010; 375(9717): 834–845. 10.1016/S0140-6736(09)62000-6 20132979

[15] Berkley JA, Ross A, Mwangi I, Osier FH, Mohammed M, Shebbe M, et al Prognostic indicators of early and late death in children admitted to district hospital in Kenya: cohort study. BMJ. 2003; 326(7385): 361 12586667 10.1136/bmj.326.7385.361 PMC148891

[16] Laman M, Ripa P, Vince J, Tefuarani N. Can clinical signs predict hypoxaemia in Papua New Guinean children with moderate and severe pneumonia? Ann Trop Paediatr. 2005; 25(1): 23–27. 15814045 10.1179/146532805X23317

[17] Dunsmuir D, Petersen C, Karlen W, Lim J, Dumont GA, Ansermino JM. The Phone Oximeter for mobile spot-check. Anesth Analg. 2012; 115(2 Suppl): S21.

[18] Karlen W, Gan H, Chiu M, Dunsmuir D, Zhou G, Dumont GA, et al Improving the accuracy and efficiency of respiratory rate measurements in children using mobile devices. PLoS One. 2014; 9(6): e99266 10.1371/journal.pone.0099266 24919062 PMC4053345

[19] Petersen CL, Görges M, Dunsmuir D, Ansermino JM, Dumont GA. Experience report: functional programming of mHealth applications. In Proceedings of the 18th ACM SIGPLAN international conference on Functional programming. 2013; 48 (9): 357–362. 10.1145/2544174.2500615

[20] Harris PA, Taylor R, Thielke R, Payne J, Gonzalez N, Conde JG. Research electronic data capture (REDCap)—a metadata-driven methodology and workflow process for providing translational research informatics support. J Biomed Inform. 2009; 42(2): 377–381. 10.1016/j.jbi.2008.08.010 18929686 PMC2700030

[21] R Development Core Team. R: A Language and Environment for Statistical Computing. Vienna, Austria: the R Foundation for Statistical Computing 2012. ISBN: 3-900051-07-0.

[22] Benatar SR, Hewlett AM, Nunn JF. The use of iso-shunt for control of oxygen therapy. Br J Anaesth. 1973; 45(7): 711–718. 10.1093/bja/45.7.711 4581075

[23] Pencina MJ, D’Agostino RB Sr, D’Agostino RB Jr, Vasan RS. Evaluating the added predictive ability of a new marker: from area under the ROC curve to reclassification and beyond. Stat Med 2008; 27 (2): 157–172; discussion 207–212. 17569110 10.1002/sim.2929

[24] Little RJA and Rubin DB. Statistical analysis with missing data. 2nd edition Hoboken: Wiley-Interscience; 2002.

[25] Schafer JL. Multiple imputation: a primer. Stat Methods Med Res. 1999; 8(1): 3–15. 10347857 10.1177/096228029900800102

[26] Harrell FE, Lee KL, Mark DB. Multivariable prognostic models: issues in developing models, evaluating assumptions and adequacy, and measuring and reducing errors. Stat Med. 1996; 15: 361–387. 8668867 10.1002/(SICI)1097-0258(19960229)15:4<361::AID-SIM168>3.0.CO;2-4

[27] Steyerberg EW, Harrell FE Jr, Borsboom GJ, Vegouwe Y, Habbema JD. Internal validation of predictive models: efficiency of some procedures for logistic regression analysis. J Clin Epidemiol. 2001; 54(8): 774–81. 11470385 10.1016/s0895-4356(01)00341-9

[28] Janes H, Pepe MS, Gu W. Assessing the value of risk predictions by using risk stratification tables. Ann Intern Med. 2008; 149: 751–760. 19017593 10.7326/0003-4819-149-10-200811180-00009 PMC3091826

[29] Thompson M, van den Bruel A, Verbakel J, Lakhanpaul M, Haj-Hassan T, Stevens R, et al Systematic review and validation of prediction rules for identifying children with serious infections in emergency departments and urgent-access primary care. Health Technol Assess. 2012; 16(15): 1–99. 10.3310/hta16150 22452986 PMC4781278

[30] Petersen CL, Chen TP, Ansermino JM, Dumont GA. Design and evaluation of a low-cost smartphone pulse oximeter. Sensors. 2013; 13(12):16882–93. 10.3390/s131216882 24322563 PMC3892845

[31] Källander K, Tibenderana JK, Akpogheneta OJ, Strachan DL, Hill Z, ten Asbroek AH, et al Mobile health (mHealth) approaches and lessons for increased performance and retention of community health workers in low- and middle-income countries: A review. J Med Internet Res. 2013; 15(1): e17 10.2196/jmir.2130 23353680 PMC3636306

[32] Ansermino JM. Universal access to essential vital signs monitoring. Anesth Analg. 2013; 117(4): 883–890. 10.1213/ANE.0b013e3182a1f22f 24023026

[33] Spruijt B, Vergouwe Y, Nijman RG, Thompson M, Oostenbrink R. Vital signs should be maintained as continuous variables when predicting bacterial infections in febrile children. J Clin Epidemiol. 2013; 66(4): 453–7. 10.1016/j.jclinepi.2012.09.014 23306061

[34] Nijman RG, Thompson M, van Veen M, Perera R, Moll HA, Oostenbrink R. Derivation and validation of age and temperature specific reference values and centile charts to predict lower respiratory tract infection in children with fever: prospective observational study. BMJ. 2012; 345(July): e4224 10.1136/bmj.e4224 22761088 PMC3388747

[35] Onyango FE, Steinhoff MC, Wafula EM, Wariua S, Musia J, Kitonyi J. Hypoxaemia in young Kenyan children with acute lower respiratory infection. BMJ. 1993; 306(6878): 612–5. 8369033 10.1136/bmj.306.6878.612 PMC1676956

[36] West JB. Respiratory Physiology: The Essentials. 9th edition Philadelphia: Lippincott Williams and Wilkins, 2011. ISBN-13: 978–1609136406

[37] Severinghaus JW. Simple, accurate equations for human blood O2 dissociation computations. J Appl Physiol Respir Environ Exerc Physiol. 1979; 46(3):599–602. 35496 10.1152/jappl.1979.46.3.599

[38] World Health Organization. Revised WHO classification and treatment of pneumonia in children at health facilities: evidence summaries. 2014. Available: http://www.who.int/maternal_child_adolescent/documents/child-pneumonia-treatment/en/ 25535631

[39] Kalter HD, Schillinger JA, Hossain M, Burnham G, Saha S, de Wit V, et al Identifying sick children requiring referral to hospital in Bangladesh. Bull World Health Organ. 1997; 75 Suppl 1:65–75. 9529719 PMC2486991

[40] Bang AT, Reddy HM, Deshmukh MD, Baitule SB, Bang R. Neonatal and infant mortality in the ten years (1993 to 2003) of the Gadchiroli field trial: effect of home-based neonatal care. J Perinatol. 2005; 25 Suppl 1: S92–S107. 15791283 10.1038/sj.jp.7211277

