# Correction: Development and Internal Validation of a Predictive Model Including Pulse Oximetry for Hospitalization of Under-Five Children in Bangladesh

**DOI:** 10.1371/journal.pone.0147560

**Published:** 2016-01-15

**Authors:** Shahreen Raihana, Dustin Dunsmuir, Tanvir Huda, Guohai Zhou, Qazi Sadeq-ur Rahman, Ainara Garde, Md Moinuddin, Walter Karlen, Guy A. Dumont, Niranjan Kissoon, Shams El Arifeen, Charles Larson, J. Mark Ansermino

The images for Figs 2 and 3 are incorrectly switched. The image that appears as Fig 2 should be Fig 3, and the image that appears as Fig 3 should be Fig 2. The figure captions appear in the correct order. Please see the corrected Fig 2 and Fig 3 here.

**Fig 2:**
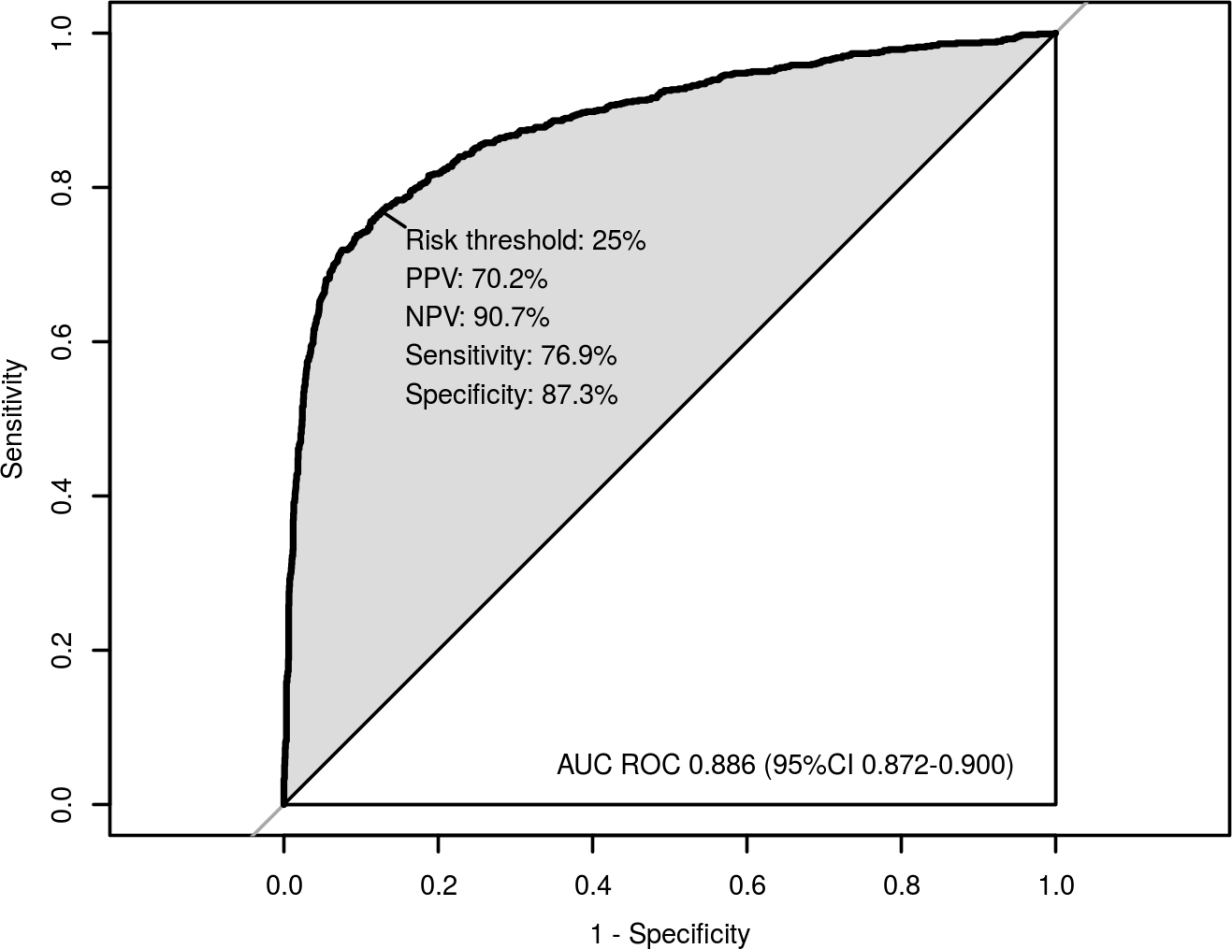
Receiver operating characteristic curve of the final model in the study cohort. AUC ROC = area under the curve of the receiver operating characteristic. Sens = sensitivity. Spec = specificity. PPV = positive predictive value. NPV = negative predictive value.

**Fig 3:**
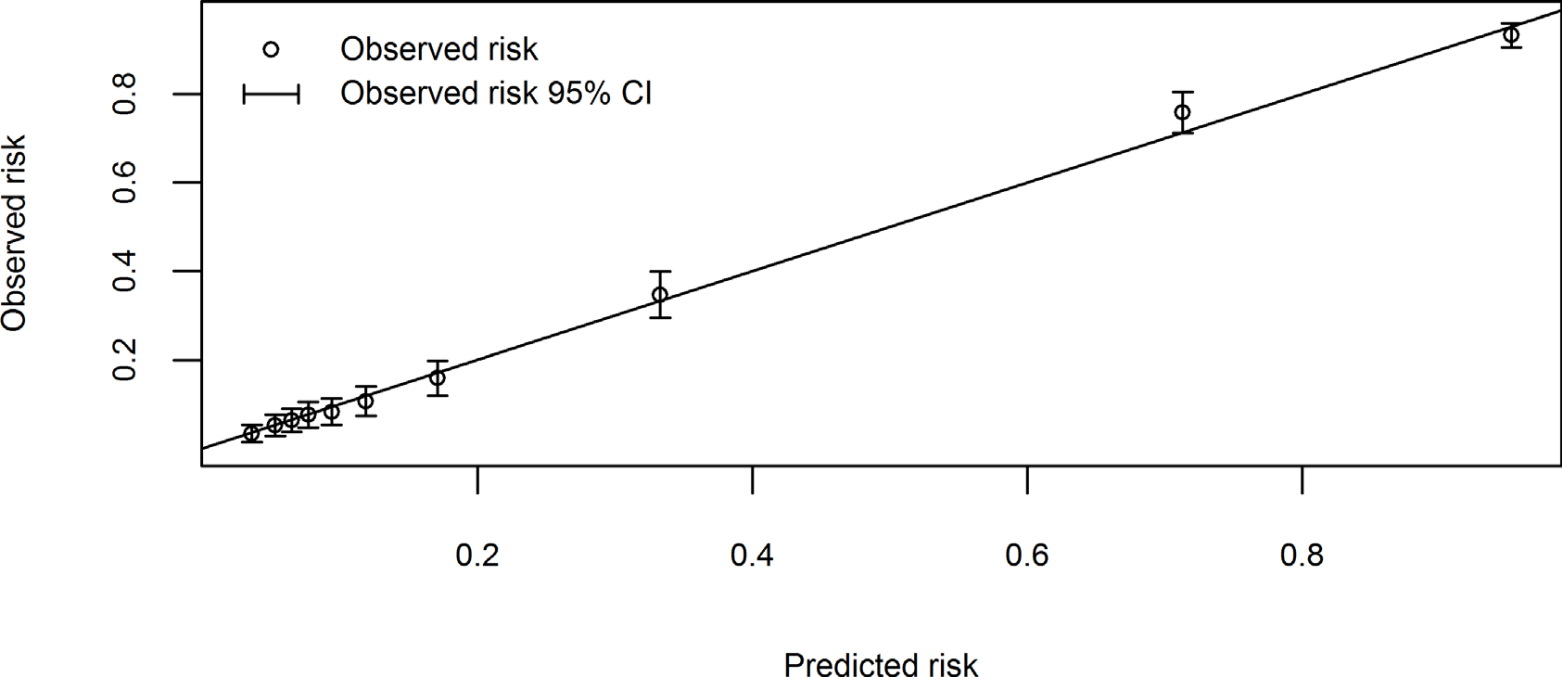
Calibration plot of the final 10-predictor model applied to the 3263 cases excluding subjects who were unconscious or who had experienced convulsions (Hosmer-Lemeshow goodness-of-fit p-value = 0.53). The 45 degree straight line corresponds to the line of perfect calibration on which model predicted risks coincide with the observed risks.
