# Supplementary material for: Development and Internal Validation of a Predictive Model Including Pulse Oximetry for Hospitalization of Under-Five Children in Bangladesh
Source: PLoS One. 2015 Nov 18;10(11):e0143213. doi: 10.1371/journal.pone.0143213 (PMC4651571; doi:10.1371/journal.pone.0143213)
Supplement: S1 File — (DOC) [file pone.0143213.s001.doc]

Appendix I: **Characteristics of children lost to follow-up**

Among the 2429 not admitted cases, we compared the followed-up and the lost to follow-up in terms of 22 characteristics as tabulated below. We applied the Mann–Whitney U test for continuous predictors and Fisher's exact test for categorical predictors. To adjust for multiple comparisons, we applied Bonferroni correction so that significance was only claimed for p-values less than 0.05/22 =0.0023.

The children lost to follow-up were on average older, had lower weight for age z-scores, a higher prevalence of fever and a lower prevalence of diarrhea. No other systematic differences were found between the children lost to follow-up and those that had been followed-up. The 596 children who we were unable to contact had all the predictors and the outcomes included in the model development and internal validation process. A further 11 children were subsequently admitted to the facility. It is possible that some of the children we were not able to contact may have presented at another facility and may have had an effect on our final conclusions.

|  |  | **Not admitted and contacted (N=1833)** | | **Not admitted and not contacted (N=596)** | | **P-value** |
| --- | --- | --- | --- | --- | --- | --- |
| **Type** | **Predictor** | **n** | **Percentage (%)** | **N** | **Percentage (%)** |  |
| **Demographic** |  |  |  |  |  |  |
|  | Gender (Male) | 1108 | 60.5 | 358 | 60.1 | 0.885 |
| **Symptoms** |  |  |  |  |  |  |
|  | Fever | 486 | 26.5 | 213 | 35.7 | <0.001 |
|  | Fever >1 day | 352 | 19.2 | 157 | 26.3 | <0.001 |
|  | Cough | 1067 | 58.2 | 346 | 58.5 | 0.962 |
|  | Cough > 1 day | 989 | 54.0 | 310 | 52.0 | 0.422 |
|  | Difficult OR fast Breathing | 113 | 6.2 | 50 | 8.4 | 0.073 |
|  | Vomiting | 187 | 10.2 | 50 | 8.4 | 0.205 |
|  | Vomiting  >1 day | 121 | 6.6 | 29 | 4.9 | 0.142 |
|  | Abdominal pain | 74 | 4.0 | 25 | 4.2 | 0.905 |
|  | Diarrhea | 346 | 18.9 | 71 | 12 | <0.001 |
|  | Diarrhea  >1day | 245 | 13.4 | 50 | 8.4 | 0.001 |
| **Signs** |  |  |  |  |  |  |
|  | Chest in-drawing | 12 | 0.7 | 6 | 1.0 | 0.410 |
|  | Difficult breathing | 25 | 1.4 | 15 | 2.5 | 0.064 |
|  | Lethargy | 99 | 5.4 | 49 | 8.2 | 0.018 |
|  | Irritability | 58 | 3.2 | 15 | 2.5 | 0.491 |
|  | Altered consciousness | 0 | 0.0 | 1 | 0.2 | 0.245 |
|  |  |  |  |  |  |  |
|  |  | **Median** | **Interquartile range** | **Median** | **Interquartile range** |  |
| **Continuous predictors** |  |  |  |  |  |  |
|  | Age in days | 409 | 192 to 565 | 474 | 245 to 632 | 0.002 |
|  | Weight for age z-score | -0.64 | -1.57 to 0.25 | -0.97 | -1.85 to 0.05 | <0.001 |
|  | Heart rate | 129 | 115 to 143 | 127 | 115 to 143 | 0.284 |
|  | Respiratory rate | 33.4 | 29.0 to 39.0 | 32.3 | 28.0 to 38.0 | 0.006 |
|  | Oxygen saturation | 98 | 97 to 99 | 98 | 97.5 to 99 | 0.016 |
|  | Temperature in ⁰Celsius | 37.0 | 36.8 to 37.0 | 37.0 | 36.8 to 37.1 | 0.139 |
